# Smartphone-Based Refractive Index Optosensing Platform Using a DVD Grating

**DOI:** 10.3390/s22030903

**Published:** 2022-01-25

**Authors:** Carlos Angulo Barrios

**Affiliations:** Department of Photonics and Bioengineering, CEMDATIC, ETSI Telecomunicación, Universidad Politécnica de Madrid, Ciudad Universitaria s/n, 28040 Madrid, Spain; carlos.angulo.barrios@upm.es

**Keywords:** smartphone, diffraction grating, optical sensor, refractive index, point-of-need

## Abstract

A low-cost, smartphone-based optical diffraction grating refractometer is demonstrated. Its principle of operation is based on the dependence of the diffraction efficiency of a DVD grating on the surrounding refractive index. The studied configuration uses the built-in LED flashlight and camera of a smartphone as a light source and a detector, respectively, to image the DVD grating diffraction pattern. No additional optical accessories, such as lenses, fibers, filters, or pinholes, are employed. The refractive index sensor exhibits a linear response in the refractive index range of 1.333–1.358 RIU (refractive index unit), with a sensitivity of 32.4 RIU^−1^ and a resolution of 2 × 10^−3^ RIU at the refractive index of water. This performance makes the proposed scheme suitable for affinity-based biosensing and a promising optosensing refractometric platform for point-of-need applications.

## 1. Introduction

Liquid refractive index (RI) sensing is required in numerous applications, including chemical analysis, biomedical diagnostics, and the food and agriculture industries [1,2,3,4,5,6]. Many of these utilizations demand on-site testing, which has increased the need for compact, robust, portable, and cost-effective RI sensing instruments. Commercially available traditional and digital handheld refractometers are typically used for this purpose. A traditional handheld refractometer is an analog instrument based on the critical angle principle [7,8,9] by which lenses and prisms project a shadow line onto a small glass reticle inside the instrument, which is then viewed by the user through a magnifying eyepiece [10,11]. Digital handheld refractometers commonly use a reflective detection system that relies on the total internal reflection [12,13,14] on a prism surface in contact with a liquid test sample [10,15]. Traditional and digital handheld refractometers can exhibit refractive index resolutions on the order of 10^−3^ and 10^−4^ RIU (refractive index unit), and their prices range from several tens to hundreds of euros.

In recent years, smartphones have drawn significant attention as analytical tools, particularly in (bio)chemical sensing [16,17,18,19], as they offer a portable and cost-effective alternative to bulky and costly instrumentation. Smartphones are ubiquitous, powerful pocket computers equipped with a built-in camera and a screen/flash, capable of data recording, analysis, and communication, which make them ideal measurement platforms for point-of-need applications. Concerning the use of smartphones for liquid refractive index sensing, Lertvachirapaiboon et al. [20] proposed a red-green, dual-color, fiber-optic surface plasmon resonance (SPR) system based on a smartphone that used an external LED light source and the smartphone CMOS camera to monitor the transmission spectrum of a fiber-optic SPR RI sensor. That sensing platform showed a quadratic response in the RI range of 1.325−1.344 and a linear response in the RI range of 1.325−1.330 with a resolution of 5.3 × 10^−4^ RIU. Later, Amloy et al. [21] demonstrated a smartphone-based critical angle refractometer for real-time refractive index monitoring by designing a specific optical coupler with a flow cell on top of the smartphone screen. In that platform, total internal reflection of incident light from the phone screen led to images that were sensitive to liquid RI and acquired from the front-facing phone camera. Such a configuration provided a linear response in the refractive index range of 1.3330−1.3575 with a resolution of 3.6 × 10^−4^ RIU.

In this paper, an alternative approach to sense fluid refractive indexes based on a smartphone platform is proposed and demonstrated. The main novelty of the present work lies in the use of a digital versatile disc (DVD) grating as an RI transducer in conjunction with a smartphone. The DVD grating diffracts incident light from the built-in LED flash of the smartphone, and the reflected diffracted light is detected by the smartphone camera. Diffraction efficiency changes due to refractive index variations of a liquid sample contacting the DVD grating can thus be imaged and converted into an analytical signal. The use of compact discs (CDs) and DVDs as low-cost dispersive optical elements in smartphone-based optochemical platforms has been reported by other authors [17,18]. However, those sensing schemes were based on the detection of spectral absorbance variations (colorimetry), which is a different sensing mechanism from that employed in this work (refractometry). In addition, unlike previous works, the configuration introduced here uses no optical components, such as lenses, filters, fibers, or pinholes, other than the flashlight and the camera of the smartphone, which facilitates its implementation and reduces its overall cost.

## 2. Materials and Methods

**Figure 1:**
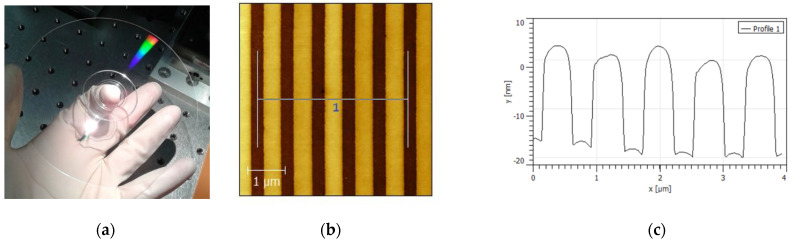
(**a**) Photograph of the DVD used as a diffraction grating for refractometric measurements. (**b**) AFM image and (**c**) profile of the surface DVD grating.

A 15 mm × 10 mm piece from a 0.6 mm-thick DVD base (without metallic and protective layers) provided by U-Tech Media Corporation (Tau-Yuan Shien, Taiwan) was used as a diffraction grating (Figure 1a). The DVD has a continuous spiral track (groove) imprinted on its plastic transparent surface, which creates a periodic structure that diffracts light. Figure 1b,c show atomic force microscopy (AFM) measurements of the DVD surface. AFM characterization reveals a surface-relief grating with a pitch of ~740 nm and a groove width and depth of ~400 nm and ~20 nm, respectively.

**Figure 2:**
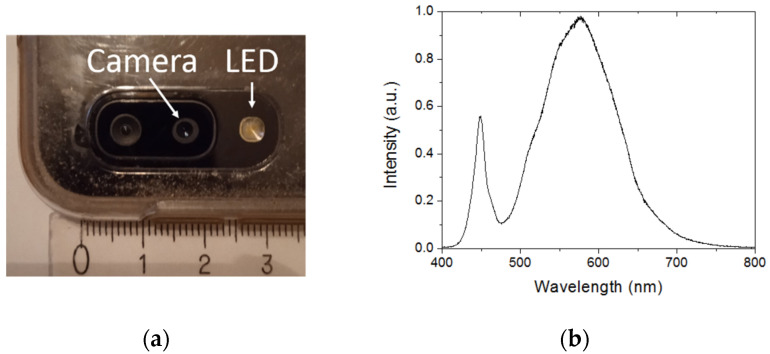
(**a**) Smartphone built-in LED flashlight and camera used in the proposed refractometric optosensing platform. The smallest division of ruler equals 1 mm. (**b**) Spectral emission of the smartphone LED flashlight.

A Samsung Galaxy A20e smartphone (Samsung, Seoul, Korea) was used for sample interrogation and measurement recording. This smartphone is equipped with an LED flashlight and a 13 MP rear camera that were employed for illuminating the DVD grating and imaging the reflected diffracted light, respectively. Figure 2a shows a photograph of the smartphone LED flashlight and camera used in this work. Figure 2b plots the spectral distribution of light emitted by the LED flashlight as measured with a spectrometer (CCS200 Thorlabs Inc., Newton, NJ, USA).

**Figure 3:**
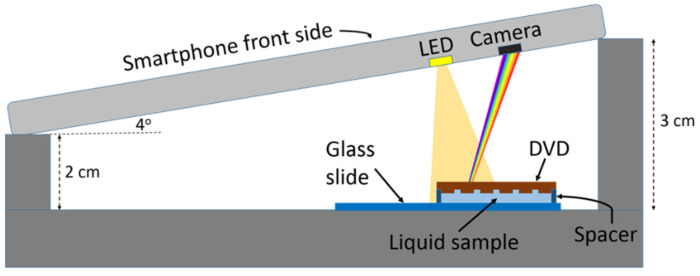
Schematic illustration of the smartphone optosensing platform for measuring the refractive index of liquid samples. Smartphone built-in LED flashlight and camera are used for illuminating a DVD grating and detecting reflected diffraction patterns, respectively. The liquid sample wets the grating surface in a fluidic cell. The diffraction intensity depends on the liquid sample refractive index.

Figure 3 shows a schematic diagram of the measurement set-up. The DVD grating was placed 25 mm away from the LED and slightly tilted at an incident angle of 4° in order to image the reflection first-order diffraction pattern. A liquid cell consisting of the DVD grating piece and a parallel glass plate, separated by 0.5 mm-thick plastic spacers, was used for sample interrogation. The DVD grating surface was facing the glass plate, and both the DVD and the glass plate were fixed to the spacers using cyanoacrylate glue. Liquid samples filled the interrogation cell by capillary action. The liquids used in the experiments were water–ethanol mixtures, with ethanol concentrations ranging from 0 to 50% (*v*/*v*), and an index-matching fluid. Refractive indexes of water–ethanol mixtures were measured with an Abbe-2WAJ refractometer, whereas the refractive index of the index-matching fluid was assumed to be 1.43 (from product specifications). The fluidic cell was cleaned and dried with compressed air after each water–ethanol measurement. The experiments were conducted at room temperature in a darkroom environment.

Images of reflected diffracted light captured by the smartphone camera were recorded as JPEG files. Freeware ImageJ (National Institutes of Health, Bethesda, MD, USA) [22], run on a standard computer, was employed for image analysis. A rectangular area covering the imaged diffraction pattern was chosen as the region of interest (ROI). The recorded images were first background-corrected using the rolling ball method [22]. Then, the mean values of the red (R), green (G), and blue (B) channels and the gray value (GV) were calculated and used as the sensor response. The mean value was defined as the sum of all pixel values in the ROI divided by the number of ROI pixels, whereas the gray value was equal to (R + G + B)/3 [22].

## 3. Results

**Figure 4:**
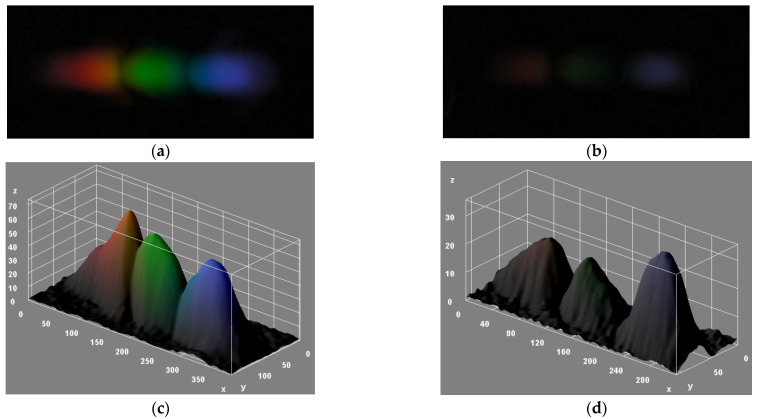
Reflected first diffraction order images from the DVD grating, recorded by the smartphone and background corrected with ImageJ, for (**a**) air and (**b**) water samples. Three-dimensional surface RGB intensity value plots of (**a**,**b**) are shown in (**c**,**d**), respectively. x-axis and y-axis units are pixels, and z-axis is scaled in pixel intensity units (ranging from 0 to 255).

Figure 4a,b show captured images, after background correction, of the reflected first-order diffraction pattern for air (RI = 1) and water (RI = 1.333), respectively. Figure 4c,d illustrate the 3D-surface RGB intensity plots of Figure 4a,b, respectively. For both fluids, spatial separation of the red, green, and blue colors produced by diffraction is clearly observed. It is also seen that the air sample produces significantly larger RGB intensity values than that corresponding to water.

**Figure 5:**
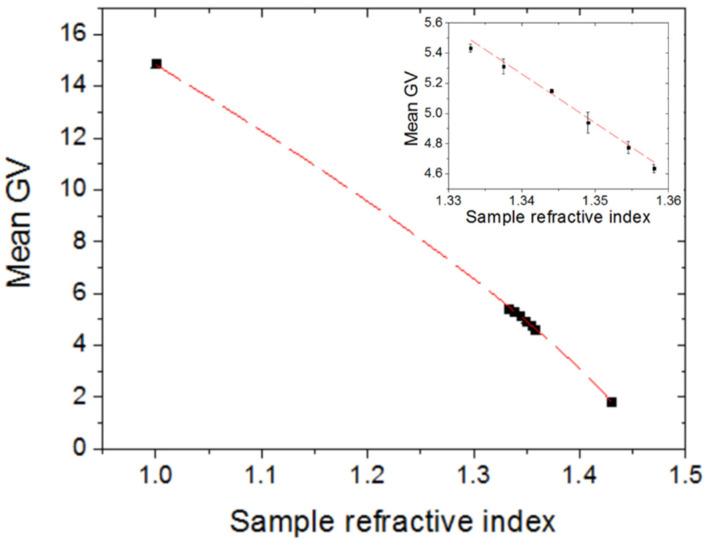
Mean gray value (GV) as a function of the sample refractive index in the interval [1, 1.43]. Inset shows the mean GV in the RI range [1.333, 1.358]. Dashed red lines represent data fits.

Figure 5 plots the measured mean GV of the diffraction pattern images as a function of the sample refractive index (n_s_) in the [1, 1.43] RI range. Each datum is the average of three successive measurements. It is seen that the mean GV decreases as the sample RI increases. The data points can be fitted by the following gamma curve (Adj R^2^ = 0.99) (the dashed red line in Figure 5):

(1) $$\mathrm{Mean}\;\mathrm{GV}=27.02\cdot {\left(1.460-n_{s}\right)}^{1/1.3}$$

**Figure 6:**
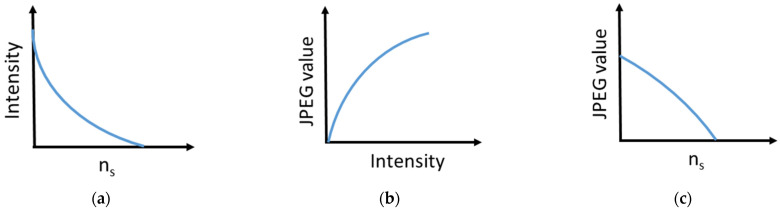
(**a**) Schematic illustrations of (**a**) the dependence of the diffracted intensity on the liquid sample refractive index (n_s_), (**b**) the dependence of the JPEG response on the light intensity (gamma correction), and (**c**) the resulting JPEG value dependence on the sample refractive index.

The first-order reflection efficiency (η), that is, the ratio of the first-order reflection optical intensity to the total incident intensity, for a thin, binary phase grating varies with the sample refractive index as [23]:

(2) $$\eta \propto {\left[\frac{\pi d\left(n_{g}-n_{s}\right)}{\lambda }\right]}^{2},$$

where d is the grating path length (i.e., the depth of the grating groove), n_g_ is the grating refractive index, and λ is the probe wavelength. Thin grating refers to the grating with a path length that is smaller than the probe wavelength. Such is the case for the employed DVD grating (d~20 nm << visible wavelengths); therefore, its diffraction efficiency should obey Equation (2), which states that η decreases quadratically with increasing n_s_. However, the measured data fit (Equation (1)) indicates a (1/1.3)th-power decrease of the diffraction intensity with increasing n_s_. This discrepancy is attributed to the non-linearity of the JPEG response exhibited by displays with the incident intensity (gamma correction) [24]. That is, JPEG value varies according to an sRGB-like profile as (incident intensity)^1/γ^, where γ is a real number. Since the intensity of the diffraction image incident on the camera should vary according to Equation (2), the resulting JPEG value should depend on the [2 × (1/γ)]th power. Thus, if 2 (1/γ) = 1/1.3, then γ = 2.6, which fits well with standard gamma values for most displays [24]. Figure 6 depicts the effect of gamma correction on the diffraction grating response.

The inset in Figure 5 shows that the sensor response can be well fitted by a linear function (Adj R^2^ = 0.98) in the [1.333, 1.358] RI interval. This RI range is particularly interesting for biosensing applications as most biological samples are water-based. The linear fit of the measured data indicates a sensitivity (S) of 32.4 RIU^−1^. The RI resolution or limit of detection (LOD) can be defined as LOD = 3σ/S [25], where σ is the standard deviation for the blank sample (pure water). From ten consecutive measurements of the mean GV for pure water, σ turned out to be equal to 0.02, which means a RI resolution of 0.002 RIU. A similar standard deviation was obtained for a series of ten consecutive measurements recorded without LED illumination, which suggests that the measurement uncertainty arises from the camera-image noise, that is, fluctuation in pixel intensity due to statistical uncertainty [26].

**Figure 7:**
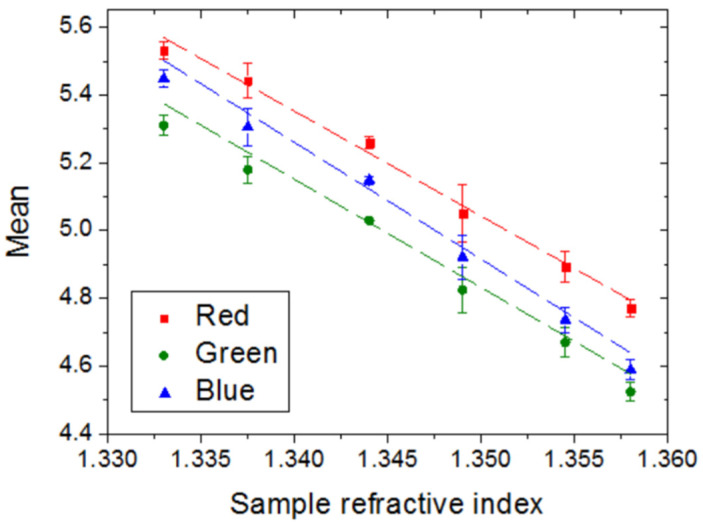
Mean value for red (red square dots), green (green circular dots), and blue (blue triangular dots) channels of the diffraction images as a function of the sample refractive index.

Figure 7 shows the measured mean red, green, and blue channel values as a function of the sample refractive index in the [1.333, 1.358] RI range. From the corresponding linear fits (dashed lines in Figure 7), the RI sensitivities for red, green, and blue signals are S_R_ = 30.9 RIU^−1^, S_G_ = 31.9 RIU^−1^ and S_B_ = 34.5 RIU^−1^, respectively. That is, the sensitivity increases as the wavelength decreases, which is in agreement with the diffraction efficiency equation (Equation (2)). Note, however, that these sensitivity values do not differ substantially from that for the mean GV. In fact, the LOD for each color channel, after rounding to one significant figure, equals 0.002 RIU, which is the same value obtained when the mean GV was considered. Therefore, either the mean of a particular color channel or the mean GV could be used as the sensor response.

## 4. Discussion

The resolution of the presented sensing configuration is larger than that reported for other smartphone-based refractometric schemes [20,21], briefly introduced in Section 1. This was expected since, unlike the aforementioned configurations, the proof of concept demonstrated here has been built with no specifically designed optical components. The RI resolution of the studied platform could be improved by using deeper DVD gratings (larger d in Equation (2)). This would increase the sample RI sensitivity of the diffraction efficiency as demonstrated experimentally in [27]. For example, a groove depth of 100 nm would enhance the diffraction efficiency sensitivity by a factor of (100/20)^2^ = 25. The fabrication of DVD gratings deeper than 20 nm should be easily attainable with standardized DVD mass-production techniques.

The performance of the proposed refractometer makes it suitable for the food, agricultural, chemical, and manufacturing industries. For example, in the quality control and management of food products, the device can be used to assess the ripeness of fruits, vegetables, sugar cane, and beets before harvesting, to check the alcohol content of wine and beer during fermentation, to measure the percentage of salt in condiments, or to assess the purity of products such as vegetable oils and animal fats. In diagnostic and veterinary medicine, the refractometer can be used to measure the total plasma protein in a blood sample and the specific gravity of urine. Note that the device operates in reflection mode; therefore, light is not required to pass through the sample where it could be absorbed. An advantage of employing reflection vs. transmission operation mode is that the former is more appropriate for use with colored or cloudy liquid samples.

It must also be noted that, according to Equation (2), a variation in the grating depth (d) can affect the diffraction efficiency similarly to changing the surrounding refractive index n_s_. In mathematical terms: Δd/d = −Δn_s_/(n_g_ − n_s_), where Δd and Δn_s_ are variations of the grating depth and liquid sample refractive index, respectively. This means that |Δn_s_| = 0.002 RIU for d = 20 nm is equivalent to |Δd| = 0.3 nm for (n_g_ − n_s_) = 0.127, where n_g_ = 1.46 (from Equation (1)) and n_s_ = 1.333 (water). Such a d variation represents the minimum variation of d that can be detected by the studied sensor in an aqueous surrounding. The thickness of a monolayer of typical biomolecular receptors, such as antibodies, antigens, and DNA molecules, is between ~5 and ~10 nm. Therefore, the demonstrated sensing platform is suitable for implementing label-free biosensing schemes that rely on the increase in the diffraction intensity of a grating produced by increments of the grating depth due to molecular binding events on the grating surface [28,29]. For the sake of illustration, numerical calculations indicated that a minimum detectable surface density of DNA molecules of 2 ng/cm^2^ could be achievable (see Appendix A for details). Such a detection limit is larger than those exhibited by high-performance plasmonic [30] and porous Si [31] biosensors based on free-space interrogation and wavelength-shift monitoring; however, it compares well to the performance of intensity-based silicon integrated optical biosensors [32,33].

Finally, concerning future works, the simplicity of the proposed platform architecture should facilitate the design and fabrication of a low-cost clip-on module for smartphones by using additive manufacturing (3D printing). Such a module would essentially consist of a plastic frame hosting the DVD-based liquid cell, which can be adapted for continuous flow operation by including proper inlet and outlet ports as, for example, reported in [21]. In addition, a smartphone software application for image analysis, such as that implemented in [20], could be developed in order to provide the analytical response directly on the smartphone screen, which would be required for actual on-site testing. Such a software application might also include a calibration procedure to be carried out before the actual sample analysis. The calibration method may consist of first measuring two reference samples: distilled water and another known refractive index aqueous solution, and then fitting the measured data by a linear function, which would provide the RI sensor sensitivity.

## 5. Conclusions

A smartphone optical platform for measuring the refractive indexes of fluids based on a DVD grating has been proposed and demonstrated. The proof-of-concept RI sensor exhibits a linear response over the RI range of 1.333−1.358 and shows a limit of detection of 0.002 RIU around the refractive index of water (1.333). This RI resolution can be translated to a grating depth resolution smaller than typical biomolecule layer thicknesses, which supports the suitability of the proposed platform for label-free biosensing based on monitoring diffraction efficiency changes due to affinity binding events on the grating surface. The studied configuration does not employ optical components (lenses, filters, fibers, or pinholes) other than the smartphone built-in functionalities, which, in addition to the use of low-cost DVDs, should contribute to the implementation of cost-saving optical designs for in-field applications.

## Figures and Tables

**Figure A1:**
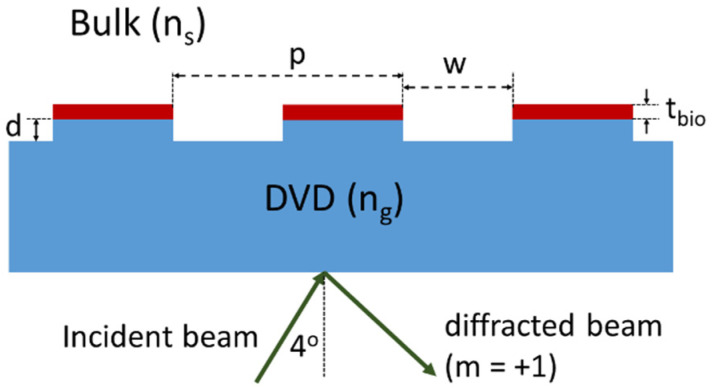
Schematic diagram of the simulated DVD grating. A biolayer of thickness t_bio_ is adhered on the top surface of the grating. Light enters from the backside of the grating at an incident angle of 4°, and the first-order diffraction efficiency is calculated by the RCWA method.

## Data Availability

Data are contained within the article.

## Appendix A

Rigorous coupled-wave analysis (RCWA) numerical calculations [34] of the first-order reflection diffraction efficiency (η) of the DVD grating were performed to evaluate the suitability of the demonstrated refractometric platform for surface biosensing.

Figure A1 shows a schematic diagram (cross-sectional view) of the simulated grating structure. Light at λ = 550 nm impinges on the grating through the DVD at an incident angle of 4°. Such a wavelength was chosen as an intermediate value of the actual white LED broad-spectrum emission. The period (p), groove width (w) and depth (d), and refractive index (n_g_) of the grating are 740 nm, 400 nm, 20 nm, and 1.46, respectively. The bulk refractive index (n_s_) equals 1.333 (water) and a DNA monolayer (thickness t_bio_ = 5 nm [35], refractive index n_bio_ = 1.43 [35]) is assumed to be adhered on the top surface of the DVD grating.

The device surface sensitivity (S_Γ_)—defined as Δη_bio_/Γ_0_, where Δη_bio_ is the difference of η with biolayer and η without biolayer, and Γ_0_ = 3.9 × 10^−14^ mol/mm^2^ [35] is the biolayer surface density — was calculated to be 1.16 × 10^9^ mm^2^/mol. Additionally, simulation of a bulk refractive index variation of Δn_s_ = 0.002 (i.e., the RI resolution of the experimental refractometric platform) without the biolayer (t_bio_ = 0) led to a diffraction efficiency variation of Δη_LOD_ = −3.4 × 10^−6^. Therefore, according to these RCWA calculations, the minimum DNA surface density detectable by the demonstrated smartphone–DVD optosensing configuration would be |Δη_LOD_|/S_Γ_ = 3.4 × 10^−6^/(1.16 × 10^9^ mm^2^/mol) = 3 × 10^−15^ mol/mm^2^, that is, 2 ng/cm^2^ (DNA molecular weight = 6281.21 Da [35]).
